# Society Meetings

**Published:** 1900-08

**Authors:** 


### SOCIETY MEETINGS.

The American Association of Obstetricians and Gynecologists will hold its thirteenth annual meeting in the Assembly room of the Galt House, Louisville, Ky., Tuesday, Wednesday and Thuısday, September 18, 19 and 20, 1900, under the presidency of Dr. Rufus Bartlett Hall, of Cincinnati, O.

The following-named papers have been offered:

1. President's address, R. B. Hall, Cincinnati.

2. Ovarian fibroma—case with microscopical report—L. H. Laidley, St. Louis.

3. Cholelithiasis—with report of cases—H. E. Hayd, Buffalo.

4. Appendicitis during pregnancy, Charles G. Cumston, Boston.

5. Diagnosis of ectopic pregnancy before rupture, based on ten cases, J. F. Baldwin, Columbus.

6. Three cases of extrauterine pregnancy, with specimens, W. B. Dorsett, St. Louis.

7. The private hospital, Joseph Price, Philadelphia.

8. Paper (Title undetermined), E. F. Fish, Milwaukee.

9. Paper (Title undetermined) C. C. Frederick, Buffalo.

10. Extirpation of the rectum and sigmoid per vaginam, John B. Murphy, Chicago.

11. Paper (Title undetermined) H. O. Pantzer, Indianapolis.

12. Paper (Title undetermined) J. H. Carstens, Detroit.

13. The hymen—of what significance is its presence or absence in determining virginity, John Milton Duff, Pittsburg.

14. Paper (Title undetermined) W. P. Manton, Detroit.

15. Paper (Title undetermined) F. Blume Pittsburg.

16. A satisfactory method for suspension of the uterus, Robert T. Morris New York.

17. Paper (Title undetermined) H. W. Longyear, Detroit.

18. Some points regarding surgery of the gall-bladder, A. Vander Veer, Albany.

19. Surgery of the liver and bile ducts, W. G. Macdonald, Albany,

20. Observations respecting malignant disease of pelvic organs. Augustus P. Clarke, Cambridge.

21. Paper (Title undetermined) M. Rosenwasser, Cleveland.

22. Bilateral celiotomy and shortening of the round ligaments for complicated retroversion of the uterus, A. Goldspohn, Chicago.

23. Paper (Title undetermined) W. B. Chase, New York City.

24. Paper (Title undetermined) Charles A. L. Reed, Cincinnati.

25. Round ligament ventrosuspension of the uterus, D. Tod Gilliam, Columbus.

26. Paper (Title undetermined) L. S. McMurtry, Louisville.

The titles of papers are announced in the order of their reception. The permanent program will be classified and issued about August 25, after which date no further titles can be added or changes made in the printed program.

A cordial invitation is extended to the medical profession to attend the several scientific sessions of the association.

---

THE Mississippi Valley Medical Association will hold its twenty-sixth annual meeting Tuesday, Wednesday and Thursday, October 9-11, 1900, under the presidency of Dr. Harold N. Moyer, of Chicago. At a meeting of the executive committee, held at Atlantic City, June 6th, the following were chosen to deliver the annual addresses : Dr. I. N. Love, of St. Louis, the address in Medicine; Dr. C. A. Wheaton, of St. Paul, Minn., the address in Surgery. The mere mention of these names is guarantee sufficient that the association will hear only the best.

Negotiations are in progress by which the members of the association may obtain a one-fair rate for the round trip for this meeting. The Southeastern Passenger Association has already granted this rate, and it is believed the Western and Central Passenger Association will concur. Due notice of this will be sent later.

It is earnestly requested that those desiring to read papers will send their titles to the secretary within the next thirty days. It is also requested that a brief synopsis of the paper of not less than twenty-five words accompany the title. Dr. Henry E. Tuley, 111 West Kentucky Street, Louisville, Ky., is the secretary.

---

THE Buffalo Academy of Medicine requests all persons desiring to contribute to the program for the sessions of 1900-1901, to communicate with the officers of sections, as announced below, on or about August 1, 1900:

Section of Medicine—Eli H. Long, chairman; Julius Ullman, secretary, 400 Franklin Street.

Section of Surgery.—Marshall Clinton, chairman; Frederick H. Millener, secretary, 5 N. Pearl Street.

Section of Obstetrics.—Albert J. Colton, chairman; William G. Ring, secretary, 364 Niagara Street.

Section of Pathology—Eugene A. Smith, chairman; F. W. McGuire, secretary, 1540 Main Street.

Section of Ophthalmology, Otology, etc.—F. W. Hinkel, chairman; George F. Cott, secretary, 531 Mooney-Brisbane Building.

It should be remembered that a prize of fifty dollars is offered by Dr. Marcel Hartwig, president of the Academy, for the best paper presented to the Academy, by a Fellow thereof, during the session 1900-1901.

